# Adolescent Sport Participation and Age at Menarche in Relation to Midlife Body Composition, Bone Mineral Density, Fitness, and Physical Activity

**DOI:** 10.3390/jcm9123797

**Published:** 2020-11-24

**Authors:** Suvi Ravi, Urho M. Kujala, Tuija H. Tammelin, Mirja Hirvensalo, Vuokko Kovanen, Maarit Valtonen, Benjamin Waller, Pauliina Aukee, Sarianna Sipilä, Eija K. Laakkonen

**Affiliations:** 1 Faculty of Sport and Health Sciences, University of Jyväskylä, 40014 Jyväskylä, Finland; urho.m.kujala@jyu.fi (U.M.K.); mirja.hirvensalo@jyu.fi (M.H.); 2 LIKES Research Centre for Physical Activity and Health, 40700 Jyväskylä, Finland; tuija.tammelin@likes.fi; 3 Gerontology Research Center and Faculty of Sport and Health Sciences, University of Jyväskylä, 40014 Jyväskylä, Finland; vukekovanen@gmail.com (V.K.); sarianna.sipila@jyu.fi (S.S.); eija.k.laakkonen@jyu.fi (E.K.L.); 4 Research Institute for Olympic Sports, 40700 Jyväskylä, Finland; maarit.valtonen@kihu.fi; 5 Physical Activity, Physical Education, Sport and Health Research Centre, Sports Science Department, School of Social Sciences, Reykjavik University, 102 Reykjavik, Iceland; benw@ru.is; 6 Department of Obstetrics and Gynecology, Pelvic Floor Research and Therapy Unit, Central Finland Central Hospital, 40620 Jyväskylä, Finland; pauliina.aukee@ksshp.fi

**Keywords:** adolescent athlete, female athlete, age at menarche, body composition, bone mineral density, physical performance, physical activity

## Abstract

This study aimed to investigate the associations of competitive sport participation in adolescence and age at menarche (AAM) with body composition, femoral neck bone mineral density (BMD), physical performance, and physical activity (PA) in middle-aged women. 1098 women aged 47–55 years formed the sample of this retrospective study. Participants self-reported their PA level at age 13–16 years and AAM. The protocol also included dual-energy X-ray absorptiometry, physical performance tests, and accelerometer-measured PA. Participants were divided into three groups according to their PA level at the age of 13–16 (no exercise, regular PA, and competitive sport) and according to their AAM (≤12, 13, and ≥14 years). After adjusting for potential confounding factors, participation in competitive sport at age 13–16 was associated with higher midlife lean mass and BMD, and better physical performance compared to groups with no exercise or regular PA. Individuals with AAM ≥ 14 years had lower midlife BMI and fat mass than participants in the other AAM groups and pre- and perimenopausal women with AAM ≥ 14 years had lower BMD than those with AAM ≤ 12. The findings indicate that participation in competitive sport in adolescence is associated with healthier body composition, higher BMD, and better physical performance in midlife, but BMD might be impaired if menarche occurs late.

## 1. Introduction

Physical activity (PA) provides many health benefits, especially during adolescence [1]. Adolescence is a critical time for skeletal growth and mineralization [2], which is enhanced with weight-bearing PA [3]. Studies with female participants have shown that sport participation in childhood/adolescence is associated with better bone health in early [4,5,6] and late adulthood [7,8,9], and former athletes have more lean mass and less fat mass than their sedentary controls in midlife [7]. PA tracks from adolescence to adulthood among women, although only at low to moderate level [10,11,12] and organised sport participation in adolescence is associated with healthy lifestyle habits later in life [13]. Despite these positive effects of PA, some female athletes suffer from conditions such as low energy availability, eating disorders or disordered eating, menstrual dysfunction and low bone mineral density (BMD), which can have adverse long-term health effects [14,15]. Further, athletes may be exposed to injuries, and consequently to later life pain and/or disabilities [16,17,18,19], but data on the prevalence of musculoskeletal problems in former female athletes compared to the general population is limited. There is also a lack of knowledge regarding fractures in former female athletes compared to non-athletes [20].

In addition to sport participation in adolescence, age at menarche (AAM) has been shown to be associated with bone health. AAM is associated negatively with BMD [21,22,23,24] and positively with fracture risk later in life [25,26]. Although female athletes tend to attain menarche later than their non-athletic peers [27,28,29], investigating the association between AAM and bone health in adulthood rarely take into account adolescence PA. Moreover, while there is evidence that AAM is inversely associated with fat percentage and BMI in adulthood [30,31,32], it is not known if AAM is associated with lean mass and physical performance in midlife.

Since, to our knowledge, no previous study has investigated long-term consequences of both sport participation in adolescence and AAM on midlife characteristics, further information on these links would provide important information for understanding these relationships. Thus, with this retrospective descriptive study, we aimed to investigate the associations between competitive sport participation in adolescence (i.e., at age 13–16) and AAM with middle-aged body composition, BMD, physical performance, and PA in 47–55-year-old women. Furthermore, we investigated if the lifetime occurrence of fractures, current musculoskeletal problems, and the lifetime occurrence of anorexia nervosa differed until middle age according to adolescent physical activity status or AAM.

## 2. Materials and Methods

### 2.1. Participants

**Figure 1:**
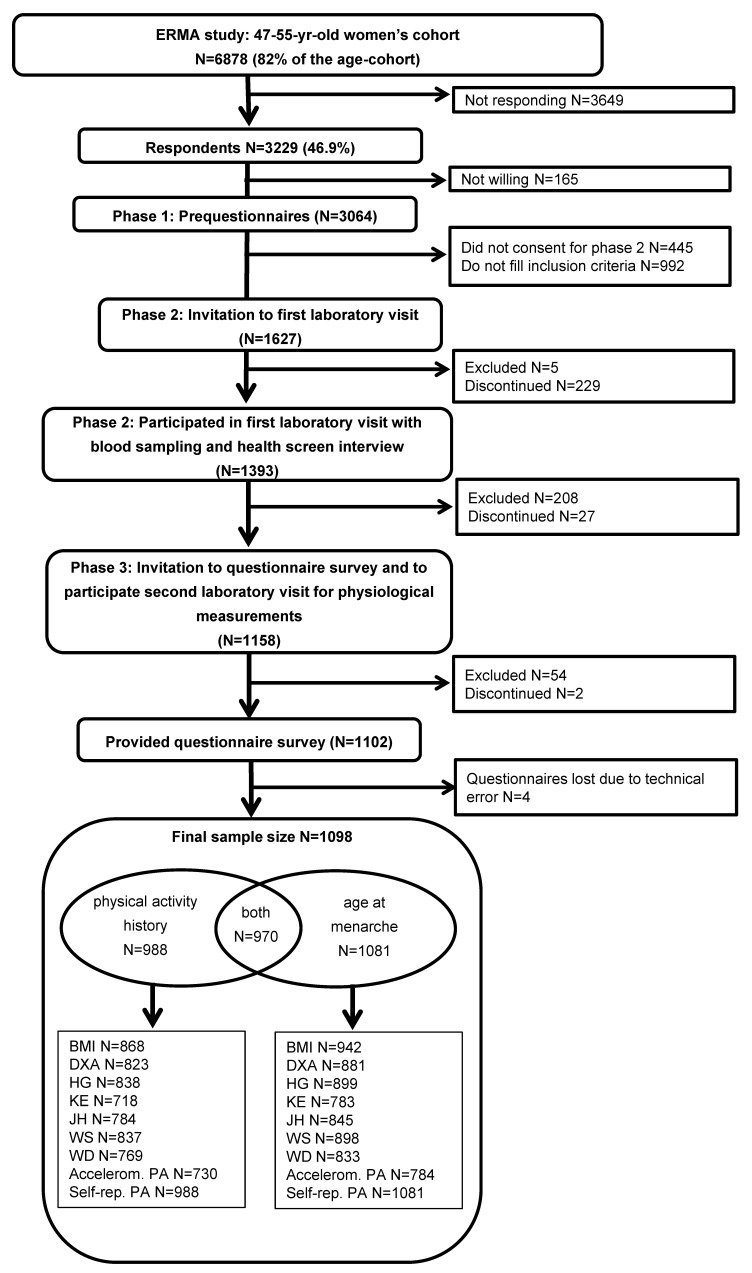
Flow chart of the recruitment of the participants. BMI = body mass index; DXA = Dual-energy X-ray absorptiometry; HG = hand grip force; KE = knee extension force; JH = jumping height; WS = walking speed; WD = walking distance; accelerom. PA = accelerometer-measured physical activity; self-rep. PA = self-reported physical activity. Some of the participants were excluded for some or all physical performance tests for the health reasons.

In the current study, data collected in the Estrogenic Regulation of Muscle Apoptosis (ERMA) study [33] were utilised. A written invitation was sent to 6878 women representing a random sample of 47–55-year-old women living in Jyväskylä or neighbouring municipalities in Central Finland. The response rate was 47%. From the eligible participants (*n* = 1627), 1393 gave blood samples and 1102 of them provided full questionnaire data. Due to technical error, four questionnaires were lost. Thus, the sample size of the present study was 1098. Of the whole sample, 988 provided information about their physical activity (PA) level at age 13–16 and 1081 responded to the question regarding age at menarche (AAM) (Figure 1).

In the ERMA study, the exclusion criteria were conditions and use of medications affecting ovarian function, obesity (self-reported BMI > 35 kg/m^2^), or conditions hindering daily physical or mental functioning.

The study was approved by the Ethics Committee of the Central Finland Health Care District (K-S shp Dnro 8U/2014) and all the participants provided written informed consent. The study protocol is described in detail in Kovanen et al. [33].

### 2.2. Assessment Methods

#### 2.2.1. Adolescent Predictors

##### PA Level

Participants described their PA level at age 13–16 as one of four possible options: no exercise, regular independent leisure-time PA, regular other supervised PA in a sport club etc., and regular competitive sport and training related to that sport (modified by Hirvensalo et al. [34]). Regular independent leisure-time PA was defined as regular PA during a journey to or from school/work (>2 km/one way) or as regular PA causing sweating that is not organised by a school, sports club, fitness centre, etc.

The definition for supervised PA was as follows: all regular non-competitive PA, organised by a sports club, fitness centre, Girl Scouts, etc. Competitive sport and related training was defined as regular, goal-oriented competitive sport within a sports club etc., and competing and training in that discipline.

Participants were classified into three groups based on their answers: no exercise group, regular PA group, which includes participants who reported performing independent or supervised or both but did not report being competing in any sport, and competitive sport group. Participants were also asked what sports disciplines they were engaged in and they were instructed to underline the disciplines in which they competed. PA level at other age stages 7–12, 17–19, 20–29, 30–39, and 40–50 years was similarly assessed to show the tracking of PA.

##### AAM

Participants self-reported the age of their first menstruation (age at menarche; AAM) and were subsequently categorised in approximately equal groups as follows: ≤12 years, 13 years, and ≥14 years.

#### 2.2.2. Middle Age Characteristics

##### Anthropometrics and Body Composition

All of the anthropometrics and body composition measurements were assessed between 7:00 and 10:00 a.m. after overnight fasting. Participants’ weight was measured in their underwear to the nearest 0.01 kg with a beam scale. Height was measured to the nearest 1.0 cm with a stadiometer. BMI was calculated as weight in kilograms divided by height in meters squared. Femoral neck BMD and body composition were assessed by dual x-ray absorptiometry (DXA, LUNAR; GE Healthcare, Chicago, IL, USA) [35]. Fat percentage was calculated as total fat mass divided by total mass multiplied by 100. Fat and lean mass indexes were calculated as fat mass (kg) and lean mass (kg), respectively, divided by height squared (m^2^). Appendicular lean mass index (ALMI) was calculated as arms lean mass (kg) plus legs lean mass (kg) divided by height squared (m^2^).

##### Physical Performance Tests

Muscle performance measurements were performed as presented earlier in Bondarev et al. 2018 [36]. Briefly, hand grip force was assessed on the dominant arm in a sitting position with the elbow flexed at 90° angle using an adjustable dynamometer chair (Good Strength, Metitur, Jyväskylä, Finland). Participants were instructed to squeeze the handle as forcefully as possible and maintain the contraction for 2–3 s. Maximal isometric knee extension force was measured in a sitting position, knee fixed in 60 degrees of flexion, on a custom-made dynamometer chair (Good Strength; Metitur Oy, Palokka, Finland) from the side of the dominant hand. Participants were instructed to extend the knee towards full extension to produce maximal force. In both the abovementioned tests, the trunk was stabilized to exclude the compensation of other muscles. Lower body muscle power was assessed with a countermovement jump performed on a custom-made contact mat. Flight time (t) was recorded and vertical jumping height (cm) was calculated as (*g* × t^2^) / 8 × 100, where *g* is the acceleration of gravity (9.81 m/s^2^) [37]. In all of the three aforementioned muscle force and power tests, three to five maximal efforts were performed, and the highest value was recorded.

Walking speed was assessed via a 10-m walk in a laboratory corridor using photocells, with five meters allowed for acceleration prior to measurement [38]. Participants performed two trials with the fastest accepted as their final result. The 6-min walking test was performed on a 20-m indoor track and the participants were instructed to walk as many laps as possible in 6 min. This test was used to assess submaximal exercise tolerance and aerobic capacity [39]. The rest between the different physical performance tests and between repetitions were separated by an approximately one-minute pause. The same protocol was applied in every participant (i.e., the instructions for the second tests were given in the similar way) resulting in approximately similar time between the tests in each participant.

##### Accelerometer-Measured PA

Accelerometer-measured PA was assessed using GT3X+ and wGT3X+ accelerometers (ActiGraph, Pensacola, FL, USA) [40]. Participants were instructed to wear the accelerometer on their right hip during waking hours (excluding activities in water) for seven consecutive days. Each participant was personally individually instructed regarding the use of the accelerometer and was also provided with a diary accompanied with the instructions to record their wake-up time, working hours, and periods when the accelerometer was removed for over 30 min. Raw acceleration data were collected at 60 Hz and consequently filtered and converted into 60 s epoch counts. Further data analysis was conducted with Excel-based customised program. Tri-axial vector magnitude cut-off -points of moderate and vigorous physical activity were >2690 to ≤6166 cpm and >6166 cpm, respectively [40,41]. The mean times spent in moderate and vigorous activity per day were summed to obtain the total mean time spent in moderate-to-vigorous physical activity (MVPA) per day. The mean daily step count was also documented.

##### Self-Reported PA

Self-reported PA was inquired using a series of modified structured questions [33,42]. Recently, this tool has been compared with accelerometer-based data in this same cohort and has found to demonstrate similar association between accelerometer-measured PA compared to other widely used self-reported PA questionnaires [43]. Participants were asked about their leisure physical activity, including monthly frequency, mean duration, and mean intensity of PA sessions; and PA during the journey to and from work. Volume of activity was calculated using an activity metabolic equivalent (MET) index by assigning a multiple of resting metabolic rate (MET score) to each activity and calculating the product of intensity × duration × frequency of activity [42].

The following MET values were used (work metabolic rate divided by resting metabolic rate): 4 (for exercise intensity corresponding to walking and for physical activity during the work journey), 6 (vigorous walking to jogging), 10 (jogging), and 13 (running) [44]. The activity MET index was expressed as the score of leisure MET hours per day and has been validated by Waller et al. [45].

##### Background Variables

Participants reported their level of education by choosing from eight answer options of primary school to doctoral level. Participants were classified into two groups based on their answers: those with bachelor level or higher education and those with education lower than bachelor level.

Participants also reported their number of live births, if they had ever used oral contraceptive pills and in what year they had started and finished using them. Participants also reported use of hormonal contraceptives (HC) during the preceding 10 years.

The menopausal status determination procedure followed the Stages of Reproductive Aging Workshop +10 guidelines [46]. Participants kept a menstrual diary for 6–12 months for menstrual cycle assessment. Serum samples to measure FSH concentrations were obtained during menstrual days 1–5 for all women who had detectable menstrual bleeding at the onset of study. For participants who had undergone hysterectomy or were using progesterone-containing contraceptives, the menopausal group assignment was based solely on the FSH level, but with stricter cut-off values than used for participants with natural menstrual bleeding [33].

Participants were also asked if they have had one or more fractures in their wrist or forearm, tibia, femur, or at some other location. Participants were also questioned if they had some musculoskeletal disorders or symptoms. Additionally, participants were asked to report if they had ever had a diagnosis of anorexia nervosa and in which year the diagnosis was given.

### 2.3. Statistical Analysis

All the continuous variables were tested for normality prior to statistical analysis. Continuous variables are presented as means and 95% confidence intervals, and categorical variables as frequencies and percentages. Differences between groups were investigated using one-way analysis of variance (ANOVA) with Bonferroni post hoc pairwise comparisons or Kruskal-Wallis rank sum test with subsequent pairwise comparisons for continuous variables, and Pearson’s chi-square for categorical variables. Analysis of covariance (ANCOVA) was used to adjust for potential confounding factors (self-reported leisure-time PA in adolescence and in midlife, age, AAM, menopausal status, number of parities, education, and HC use during the 10 preceding years). As we assumed that BMD in postmenopausal women regardless of AAM may be lower than in women within the other menopausal status groups, and therefore potentially skew results or cloud an association between AAM and BMD, we investigated the association between AAM and BMD also in pre-, early perimenopausal, and late perimenopausal women only. Statistical analyses were conducted using IBM SPSS Statistics version 24 (Armonk, NY, USA). The significance level was set at <0.05, two-tailed.

## 3. Results

### 3.1. PA Participation from Childhood to Midlife

The most common sport disciplines (during 1970s and 1980s) in the competitive sport group were track and field (28.7%), volleyball (20.6%), cross-country skiing (18.4%), running (16.9%), Finnish baseball (7.4%), and gymnastics (7.4%). Most of the participants who engaged in competitive sport at age 13–16 reported having competed in two or more sport disciplines (*n* = 98, 72.1%).

For each age stage, PA level was compared to the individual’s PA level at age 13–16 years (i.e., adolescence). At every age stage except the latest (i.e., age 40–50 years), those who engaged in competitive sport at age 13–16 years formed the larger proportion of those in competitive sport compared to those that reported regular PA or no exercise at age 13–16 years (*p* for all <0.001). Conversely, those who engaged in competitive sport at age 13–16 years formed the smaller proportion of those with no exercise at all at other age stages except the latest compared to those that reported regular PA or no exercise at age 13–16 years (*p* for all <0.001). There was no difference in the PA level groups between ages 13–16 and 40–50 (*p* = 0.272) (Table 1). The proportion of the participants engaged in competitive sport was highest at age 13–16 and gradually decreased thenceforth. Furthermore, the proportion of those reporting regular PA increased at each age stage after the age of 13–16 (Figure S1). The correlation coefficients between PA level at age 13–16 and PA level at ages 7–12, 17–19, 20–29, 30–39, and 40–50 were 0.54, 0.60, 0.29, 0.19 (*p* < 0.001 for all), and −0.02 (*p* = 0.463), respectively.

### 3.2. Midlife Characteristics According to Adolescence PA

Those who were engaged in competitive sport at age 13–16 were slightly younger and had used HC more often during the preceding 10 years than participants in the other two groups. Participants in the competitive sport group also had a higher education level in midlife and later AAM than participants with no exercise (Table 2).

Participants in the competitive sport group had more lean mass, higher lean mass index, ALMI, and BMD than those with no exercise or regular PA. They also performed better in all physical performance tests than participants in the other two groups. Self-reported PA at midlife was higher in the competitive sport group than in the other groups but no difference was found in accelerometer-measured PA between the groups. Differences in total lean mass, lean mass index, ALMI, BMD, physical performance, and self-reported leisure-time PA between the competitive sport group and the other groups remained after adjustment for potential confounding factors (Table 2). Participants who did not respond to the question regarding PA at age 13–16 (11.1% of the whole sample, *n* = 110) were similar to those who completed all variables except in hand grip force (non-responders 297.9 N vs. responders 314.7 N, *p* = 0.018) and in menopausal status (proportions of premenopausal, early perimenopausal, late perimenopausal, and postmenopausal among non-responders 30.9%, 11.8%, 11.8%, and 45.5%, respectively and among responders 27.3%, 18.7%, 19.8%, and 34.1%, respectively, *p* = 0.018).

### 3.3. AAM and Midlife Characteristics

AAM among participants ranged between 9 and 19 years (Table S1) with mean age 12.95 years (95% CI 12.9–13.0). The most common menarcheal ages were 13 (34.9%), 12 (24.1%), and 14 years (17.8%). 102 (9.3%) of the participants reported that they had used OCP at some point of their life with starting age between 16–54 years. No participants reported starting OCP use before menarche and 15 of the OCP users did not provide starting year.

The group that reported AAM ≥ 14 years included the smallest portion of participants with no exercise and the greatest portion of participants with regular PA and competitive sport at age 13–16. There was a significant linear downward trend in weight, BMI, total fat mass, fat percentage, and fat mass index across the AAM groups, all of which decreased as AAM increased. All pairwise comparisons were statistically significant except for fat percentage between the group reporting AAM ≤ 12 years and the group reporting AAM = 13 years. BMD was higher in the group reporting AAM ≤ 12 years compared to the group reporting AAM ≥ 14 years and differences in physical performance were found in jump height, with the group of AAM ≥ 14 years jumping higher than the group of AAM ≤ 12 years. Participants in the group reporting AAM ≥ 14 years took more steps than those in the other groups, but no other differences in PA between the groups were found. The differences in weight, BMI, fat mass, fat mass index, ALMI, jump height, and leisure-time step count persisted when adjusted for confounding factors (Table 3). Exclusion of postmenopausal women from this analysis demonstrated an inverse association between AAM and BMD even when adjusted for PA level at age of 13–16, self-reported leisure-time PA at midlife, age, and menopausal status (*p* = 0.040). The inverse association was found also after further adding number of parities, education, and hormonal contraceptive use during the 10 preceding years as covariates in the model (*p* = 0.041). 

16.2% (*n* = 178) of the participants reported that they have had at least one fracture during their life, while 37.6% (*n* = 413) reported having current musculoskeletal disorder/symptom. Six participants (0.5%) reported that they have received a diagnosis of anorexia nervosa, while one participant did not answer this question. Age at the time of the diagnosis varied between 13 and 39 (mean 21.5, 95% CI 10.5–32.5) and two participants had received the diagnosis before AAM. No differences were found in the amount of lifetime fractures and anorexia nervosa prevalence, or current musculoskeletal problems between the different PA level in adolescence (Table S2) or AAM groups (Table S3). The participants who did not respond to the question regarding AAM (1.5%, *n* = 17) were similar to those who did in terms of all the outcome variables.

## 4. Discussion

This is the first study to describe midlife body composition, BMD, physical performance, and PA according to PA in adolescence and AAM in middle-aged women. We found that those who had been engaged in competitive sport at age 13–16 had more lean mass, higher BMD in the femoral neck, and better physical fitness in midlife than participants with regular PA or no exercise, even when adjusted for potential confounding factors. Participants in the competitive sport group had later AAM than those with no exercise in adolescence. Furthermore, participants with later AAM had lower BMI and less fat mass than those with earlier AAM, and BMD was lower in the group with AAM ≥ 14 compared to those with AAM ≤ 12. These associations persisted when adjusted for potential confounding factors, except for the difference in BMD. However, when postmenopausal participants were excluded from this analysis, an inverse relationship between AAM and BMD was found regardless of the confounding factors.

### 4.1. PA Participation from Childhood to Midlife

We observed declining correlation coefficients between PA level at age 13–16 and PA level at other age stages with advancing age, which is consistent with a meta-analysis conducted by Craigie et al. [11] who found that the strength of tracking of PA declined when the duration of the follow-up increased. In our study, participation in competitive sport in adolescence was associated with higher self-reported leisure-time PA in middle-age, which is in line with an earlier study showing that participation in competitive sport in adolescence predicts maintenance of a high PA level in old age [34]. However, we found no association between PA in adolescence and accelerometer-measured PA in midlife. We may, however, have lacked sufficient power to detect these differences as the sample sizes were smaller in accelerometer-measured compared to self-reported PA.

### 4.2. Association between PA in Adolescence and AAM

Previous studies have stated that adolescent athletes tend to attain menarche later than their less physically active counterparts [28,29], and similarly in the present study, participants in the competitive sport group and regular PA group had later AAM compared to the no exercise group. Studies have shown that although AAM is largely modified by genes [47,48,49], it is influenced by environmental factors, including PA [50]. However, since evidence regarding an association between PA before menarche and later AAM is limited [28], it is not known if there is a causal relationship between these variables, if selection bias was present or if other behavioural differences between athletes and non-athletes explain the reported association [27,28].

### 4.3. Competitive Sport in Adolescence and Midlife Characteristics

We found no difference in fat mass between the adolescent PA groups, in contrast lower fat mass in former gymnasts, runners, and swimmers compared to controls in midlife has been reported [7,9]. The inconsistency between the studies may be due to different study population, with aesthetic and endurance athletes tending to have lower BMI than athletes in other sport disciplines [27], with our study representing a variety of sporting disciplines. Furthermore, our finding concerning higher midlife lean mass in participants in the competitive sports group is in agreement with one [7] but in contrast to another [9] study.

Consistent with some [7,9] but not all studies [51], we found that there was an association between competitive sport in adolescence and higher BMD in midlife. In our study, this association persisted after adjusting for potential confounding factors. This is contrary to the findings of Nilsson et al. [52], who reported that among women aged 75–80 years PA at age 10–30 was positively associated with BMD, but this association was not seen when adjusted for current PA. Therefore, in older women, current PA might be more strongly associated with BMD than PA in adolescence [53]. Further, and again in contrast to our findings, these authors found no association between PA at age 10–30 and current hand grip strength among women aged 75–80 [52]. These inconsistencies may be due to the fact that participants in our study were younger and PA history was studied in narrower age-range than in the study conducted by Nilsson and colleagues.

While it has been found that the risk of osteoarthritis of the hip and knee is greater in former female athletes compared to the general population [54] and that former athletes have higher risk of knee problems and higher rates of Achilles tendon problems in later life compared to controls [55], we did not find an association between sport participation in adolescence and musculoskeletal problems in middle age. As we did not ask participants what kind of musculoskeletal problems they had, it is possible that in our study, specific types of problems were more common in different groups.

In contrast to our findings, Tveit et al. found that a risk of fracture was lower among former male athletes than among controls [56]. However, we did not differentiate fractures occurred during the sport career from those occurred after retirement from sport. In addition to the fact that participants in the study conducted by Tveit et al. [56] were males, they were also older than those in our study. The incidence of fractures has been reported to be higher among older women compared to our study population [57,58] and thus, fracture rates among women in our study were relatively low.

Due to low number of anorexia nervosa cases in our study, no conclusion in terms of possible association between anorexia and PA in adolescence can be made. Other studies have suggested that eating disorders in general are more common in athletes than in non-athletes [59,60]. The lifetime prevalence of anorexia nervosa in our study was lower than recently found among middle aged women living in UK [61], young adult women living in Finland [62], and also slightly lower than in women living in U.S. [63]. Incidence of eating disorders are highest in adolescence and in young adulthood [64], and our participants were at that age in 1970s and 1980s. It has been reported that anorexia nervosa has become more common since that time [65], which may explain the differences between the results of abovementioned studies and our findings. However, it is suggested that the increased prevalence rates may be due to increased detection of anorexia nervosa rather than true increase of incidence rates [65]. Thus, there might have been some undiagnosed anorexia nervosa cases among our study population as well. Furthermore, in our study, diagnosis of anorexia nervosa was self-reported and asked by a single question, which may further underestimate the actual prevalence rate.

### 4.4. AAM and Midlife Characteristics

In line with previous observational evidence [31,32,66,67,68,69,70,71,72,73], our results show that AAM is inversely associated with BMI, fat mass, and fat percentage in middle-age. The inverse relationship between AAM and BMI in adulthood was also recently supported by Mendelian randomisation studies [48,74,75]. Fewer studies have investigated the association between AAM and midlife lean mass. Similar to our finding, Kirchengast et al. [66] did not find an association between AAM and lean mass either in pre- or postmenopausal women.

The observed inverse relationship between AAM and midlife BMD in the current study is consistent with findings of some studies conducted in women with a mean age over 40 years [21,76] and also in postmenopausal women only [22,24,77]. However, not all studies have found this association [78,79,80,81,82,83,84] and also in our study, the relationship was abolished after adjusting models for potential confounding factors. While shared genetic factors have shown to influence both AAM and BMD [76,85,86], postmenopausal bone loss may interfere with this relationship; the association between early menarche and higher BMD may be stronger in premenopausal than in postmenopausal women [85]. Our finding showing a relationship, regardless of confounding factors but excluding postmenopausal women, between AAM and midlife femoral neck BMD in pre- and perimenopausal supports this hypothesis. However, higher body mass is known to associate with higher BMD [87,88], which also may affect the associations between AAM and BMD that were found in our study. Therefore, further studies are needed to clarify the effects of AAM on midlife BMD. In our study population, there were few participants with extremely late AAM (e.g., >16 years) and thus, there was no statistical power to compare that group with earlier AAM. Thus, more research is needed to investigate if there is even greater difference in midlife BMD between former athletes with the current criterion of primary amenorrhea (i.e., menarche occurred at the age of 15 or older) and those with AAM < 15 years.

In contrast to our findings, late AAM has been linked to increased fracture risk in later life [26,89,90,91,92]. A possible explanation for the controversy between earlier studies compared to ours may be that in our study participants were younger than those in the studies demonstrating this association. Furthermore, again in contrast to our findings, it has been suggested that early menarche is associated with chronic widespread musculoskeletal complaints in later life [93]. No conclusion regarding the association between anorexia nervosa and AAM can be made based on the current study due to only few anorexia nervosa cases in our study population. However, in several other studies anorexia nervosa has been linked to later menarche [94].

### 4.5. Limitations

We acknowledge some limitations. PA level in adolescence and AAM were self-reported retrospectively and thus susceptible to response or recall bias. It is reasonable to assume that those who had been competitive athletes in their adolescence did not have difficulties in assigning themselves in an accurate PA category. However, differentiation between no exercise and regular PA may have been overlapping with the possibility that participants had different perceptions for PA. In addition, it has been reported that long-term recall of AAM is fairly valid [95,96,97,98] and that the accuracy of recall is increased when AAM is categorised into groups [98]. We did not ask the precise month the participant attained menarche, which may have caused some inaccuracy; however, few middle-aged women are able to recall the precise month they started to menstruate [96,97]. Furthermore, the study design does not enable causal interpretations. Although we adjusted our analysis for potential confounding factors, residual confounding cannot be completely ruled out. It is possible that other factors, such as genetic or different lifestyle habits, affected the observed associations.

No information on menstrual history, such as history of menstrual dysfunction, was collected and thus, we do not know if the participants have had regular menses after menarche. On the other hand, recall of menstrual regularity is less accurate than recall of AAM in women near menopause [95]. In addition, we did not collect information on training volume in adolescence and thus, cannot be sure if the participants engaged in competitive sport in adolescence actually differed from those who exercised regularly in terms of training volume and intensity. Furthermore, our study sample consisted of Caucasian relatively healthy individuals with no health conditions jeopardizing physical or mental conditioning, which can limit the generalisability of the results. Finally, the sample size did not allow us to investigate interaction affects between AAM and sport participation on BMD and body composition. However, it is possible that such interactions exist. Thus, based on our results, we cannot say if, for example, individuals who participated in competitive sport during adolescence but had an AAM ≤ 12 years have better midlife BMD than those in a competitive sport group with an AAM ≥ 14 years.

It is also important to bear in mind that our participants engaged in competitive sport in their adolescence, approximately 40 years ago. Since then, sport specialisation at early age has increased globally [99] and participation in organised sport club has become more frequent in Finnish girls [100]. Early sport specialisation can lead to physical and mental harm as it has been linked to reduced motor skill development, increased injury rates, sport dropout, burnout, eating disorders, and menstrual dysfunction [99,101,102,103]. Thus, generalisability of our results to those who are currently engaged in competitive sport should be done with caution.

## 5. Conclusions

This large, retrospective study suggests that participation in competitive sport at age 13–16 is associated with higher midlife lean mass and femoral neck BMD, and better physical performance compared to regular PA or no exercise during adolescence. In addition, we found that competitive sport at age 13–16 was associated with later menarche compared to no exercise at that age. The current study also showed that AAM was negatively associated with midlife BMI and fat mass regardless of confounding factors in all participants, and with femoral neck BMD in pre- and perimenopausal women. Thus, competitive sport in adolescence seems to be associated with healthier body composition, higher BMD, and better performance in midlife, but late AAM might be associated with low BMD. Further research is needed to investigate whether middle-aged or older individuals with primary amenorrhea (i.e., AAM ≥ 15 years) differ from individual with AAM < 15 years especially in terms of BMD or fractures. Furthermore, future studies should investigate what is the volume and intensity of the exercise needed in adolescence with respect to positive outcomes in middle age seen in our study.

## Figures and Tables

**Table 1:** Proportion (*n*) of participants of different physical activity (PA) level at each age stages according to the PA level at the age of 13–16.

	Competitive Sport (CS)			Regular PA (RPA)			No Exercise (NE)		
	at Age 13–16 (*n* = 136)			at Age 13–16 (*n* = 689)			at Age 13–16 (*n* = 163)		
Age	CS	RPA	NE	CS	RPA	NE	CS	RPA	NE
7–12	50.7% (69)	45.6% (62)	3.7% (5)	5.8% (40)	85.5% (589)	8.7% (60)	3.7% (6)	36.8% (60)	59.5% (97)
17–19	48.5% (66)	47.1% (64)	4.4% (6)	2.0% (14)	88.0% (606)	10.0% (69)	1.8% (3)	29.4% (48)	68.7% (112)
20–29	22.8% (31)	70.6% (96)	6.6% (9)	2.0% (14)	87.2% (601)	10.7% (74)	2.5% (4)	65.6% (107)	31.9% (52)
30–39	8.8% (12)	85.3% (116)	5.9% (8)	1.6% (11)	89.3% (615)	9.1% (63)	0.6% (1)	76.7% (125)	22.7% (37)
40–50	1.5% (2)	91.2% (124)	7.4% (10)	1.2% (8)	94.0% (647)	4.9% (34)	0.6% (1)	95.1% (155)	4.3% (7)

**Table 2:** Midlife characteristics of participants according to physical activity (PA) level at the age of 13–16.

Variable	*n*	Competitive Sport	*n*	Regular PA	*n*	No Exercise	*p*-Value	Model 1: *p*-Value ^a,b^	Model 2: *p*-Value ^c,d^
Background variables									
Age (y)	136	50.9 (50.5–51.2) ^R,N^	689	51.4 (51.3–51.6)	163	51.6 (51.3–51.9)	**0.005** ^e^		
Age at menarche (y)	134		677		162		**0.010**		
≤12		35.1% (47) ^N^		35.2% (238) ^N^		38.9% (63)			
13		30.6% (41)		34.3% (232)		43.2% (70)			
≥14		34.3% (46)		30.6% (207)		17.9% (29)			
Bachelor or higher education	136	48.5% (66) ^N^	689	41.5% (286)	163	34.4% (56)	**0.045**		
Number of parities	136	2.0 (1.8–2.1)	687	2.1 (2.0–2.2) ^N^	163	1.9 (1.7–2.1)	**0.017** ^f^		
Used OCP at some point of life	136	6.6% (9)	689	9.9% (68)	163	11.0% (18)	0.399		
Used HC during the preceding 10 years	136	62.5% (85) ^R,N^	689	50.8% (350)	163	49.1% (80)	**0.031**		
Menopausal status	136		689		161		0.893		
PRE		30.1% (41)		27.4% (189)		24.5% (40)			
EPM		19.1% (26)		18.6% (128		19.0% (31)			
LPM		LPM 21.3% (29)		19.3% (133)		20.9% (34)			
POST		POST 29.4% (40)		34.7% (239)		35.6% (58)			
Body composition									
Height (cm)	113	166.4 (165.4–167.4)	609	165.6 (165.1–166.0)	146	164.7 (163.8–165.7)	0.074 ^e^	0.188 ^a^	0.257 ^c^
Weight (m)	113	70.3 (68.3–72.3)	609	70.3 (69.4–71.2)	146	68.5 (66.8–70.1)	0.182 ^e^	**0.046** ^a^	**0.038** ^c^
BMI (kg/m^2^)	113	25.4 (24.7–26.1)	609	25.6 (25.3–25.9)	146	25.2 (24.7–25.8)	0.431 ^e^	0.151 ^a^	0.124 ^c^
Total fat mass (kg)	101	24.0 (22.4–25.5)	584	25.5 (24.8–26.2)	138	24.4 (23.1–25.7)	0.141 ^e^	**0.041** ^a^	**0.037** ^c^
Fat percentage (%)	101	33.3 (31.8–34.7) ^R^	584	35.4 (34.8–36.0)	138	35.0 (33.8–36.2)	**0.028** ^e^	**0.022** ^a^	**0.026** ^c^
Fat mass index (kg/m^2^)	101	8.7 (8.1–9.3)	584	9.3 (9.0–9.5)	138	9.0 (8.5–9.7)	0.143 ^e^	0.065 ^a^	0.062 ^c^
Total lean mass (kg)	101	43.7 (42.8–44.6) ^R,N^	584	42.1 (41.7–42.4)	138	41.4 (40.7–42.1)	**<0.001** ^e^	**0.001** ^a^	**0.001** ^c^
Lean mass index (kg/m^2^)	101	15.8 (15.5–16.0) ^R,N^	584	15.3 (15.2–15.4)	138	15.2 (15.0–15.5)	**0.002** ^e^	**0.002** ^a^	**0.001** ^c^
ALMI (kg/m^2^)	101	6.9 (6.7–7.0) ^R,N^	584	6.6 (6.6–6.7)	138	6.6 (6.5–6.7)	**<0.001** ^e^	**0.001** ^a^	**0.001** ^c^
*BMD*									
FN BMD (g/cm^2^)	101	1.00 (0.98–1.03) ^R,N^	584	0.96 (0.95–0.97)	138	0.95 (0.93–0.97)	**<0.001** ^e^	**0.001** ^a^	**0.002** ^c^
FN T score < −1	101	14.9% (15) ^R,N^	584	25.7% (150)	138	28.3% (39)	**0.039**		
FN T score ≤ −2.5	101	0.0% (0)	584	0.9% (5)	138	0.7% (1)	1.000		
*Physical performance*									
Hand grip force (N)	106	333.5 (322.6–344.4) ^R,N^	590	313.5 (308.7–318.4)	142	305.5 (296.4–314.5)	**0.001** ^e^	**0.002** ^a^	**0.002** ^c^
Knee extension force (N)	92	509.1 (488.4–529.8) ^R,N^	508	460.6 (452.7–468.4)	118	442.3 (425.6–458.9)	**<0.001** ^e^	**<0.001** ^a^	**<0.001** ^c^
Jumping height (cm)	101	21.3 (20.5–22.1) ^R,N^	547	19.0 (18.6–19.3)	136	18.7 (18.0–19.4)	**<0.001** ^e^	**<0.001** ^a^	**<0.001** ^c^
Walking speed (m/s)	106	2.9 (2.8–3.0) ^R,N^	591	2.6 (2.6–2.7)	140	2.6 (2.5–2.6)	**<0.001** ^e^	**<0.001** ^a^	**<0.001** ^c^
Walking distance in 6 min (m)	95	696.7 (684.8–708.5) ^R,N^	543	667.5 (662.3–672.7)	131	660.7 (651.1–670.2)	**<0.001** ^e^	**<0.001** ^a^	**0.001** ^c^
*Self-reported PA*									
Leisure-time PA (MET-h/d)	136	4.9 (4.2–5.6) ^R,N^	689	4.2 (3.9–4.4)	163	3.6 (3.2–4.0)	**<0.001** ^f^	**0.008** ^b^	**0.007** ^d^
*Accelerometer-measured PA*									
Leisure-time MVPA (min/d)	88	47.6 (41.6–53.6)	516	43.1 (41.1–45.2)	126	40.7 (36.6–44.7)	0.166 ^f^	0.178 ^b^	0.271 ^d^
Leisure-time step count (steps/d)	88	7277 (6676–7878)	516	6843 (6601–7084)	126	6763 (6334–7192)	0.339 ^e^	0.302 ^b^	0.384 ^d^
Total MVPA (min/d)	88	54.3 (47.7–60.7)	516	50.3 (48.0–52.5)	126	46.7 (42.6–50.8)	0.188 ^f^	0.220 ^b^	0.312 ^d^
Total step count (steps/d)	88	8903 (8291–9515)	516	8698 (8450–8947)	126	8510 (8087–8933)	0.646 ^f^	0.743 ^b^	0.761 ^d^

Values are presented as means (95% confidence intervals) or percentages (counts). R and N indicate significant difference (*p* < 0.05) from regular PA and no exercise groups, respectively. OCP = oral contraceptive pills; HC = hormonal contraceptive; PRE = premenopausal; EPM = early perimenopausal; LPM = late perimenopausal; POST = postmenopausal; BMI = body mass index; ALMI = appendicular lean mass index; FN = femoral neck; BMD = bone mineral density; MET = metabolic equivalent; MVPA = moderate-to-vigorous physical activity. ^a^ Adjusted for self-reported leisure-time PA in midlife, age, menarcheal age, and menopausal status. ^b^ Adjusted for age, menarcheal age, and menopausal status. ^c^ Adjusted for self-reported leisure-time PA in midlife, age, menarcheal age, menopausal status, number of parities, education, and hormonal contraceptive use during the 10 preceding years. ^d^ Adjusted for age, menarcheal age, menopausal status, number of parities, education, and hormonal contraceptive use during the 10 preceding years. ^e^ ANOVA. ^f^ Kruskal-Wallis test. Sample sizes for both adjusted models were 111, 598, and 149 for competitive sport, regular PA, and no exercise groups, respectively, in height, weight, and BMI. Corresponding sample sizes for body composition measurements and BMD were 99, 573, and 137, for physical performance measurements they were 104, 579, and 141 (hand grip force), 90, 500, and 118 (knee extension force), 99, 538, and 135 (jumping height), 104, 580, and 139 (walking speed), 93, 534, and 131 (walking distance in 6 min), and for physical activity measurements they were 134, 677, and 162 (self-reported PA) and 86, 510, and 125 (accelerometer measured PA). Statistically significant *p*-values are indicated in bold.

**Table 3:** Midlife characteristics of the participants according to different menarcheal age.

Variable	*n*	AAM ≤ 12	*n*	AAM = 13	*n*	AAM ≥ 14	*p*-Value	Model 1: *p*-Value ^a,b^	Model 2: *p*-Value ^c,d^
Background variables									
Age (y)	391	51.3 (51.1–51.5)	377	51.4 (51.2–51.6)	313	51.4 (51.2–51.6)	0.465 ^e^		
Bachelor or higher education (%)	391	41.2% (161)	377	41.9% (158)	313	40.9% (128)	0.961		
Number of parities	391	2.0 (1.9–2.2)	377	2.0 (1.9–2.1)	313	2.0 (1.9–2.2)	0.937 ^f^		
Used OCP at some point of life	391	7.9% (31) ^H^	377	7.7% (29) ^H^	313	13.1% (41)	**0.025**		
Used HC during the preceding 10 years	391	54.2% (212)	377	48.0% (181)	313	53.7% (168)	0.172		
Menopausal status	390		377		313		0.373		
PRE		24.8% (97)		29.7% (112)		29.4% (92)			
EPM		20.2% (79)		17.2% (65)		16.3% (51)			
LPM		21.7% (85)		17.2% (65)		18.2% (57)			
POST		33.2% (130)		35.8% (135)		36.1% (113)			
PA at age 13–16	346		343		281		**0.014**		
CS		13.5% (47) ^H^		12.0% (41) ^H^		16.4% (46)			
RPA		68.6% (238)		67.9% (233)		73.3% (206)			
NE		17.9% (62)		20.1% (69)		10.3% (29)			
Body composition									
Height (cm)	335	165.3 (164.7–165.9)	341	165.3 (164.7–166.0)	266	166.3 (165.6–166.9)	0.073 ^e^	0.122 ^a^	0.135 ^c^
Weight (m)	335	71.7 (70.5–72.8) ^M,H^	341	69.6 (68.4–70.7)	266	68.0 (66.8–69.3)	**<0.001** ^e^	**<0.001** ^a^	**<0.001** ^c^
BMI (kg/m^2^)	335	26.2 (25.8–26.6) ^M,H^	341	25.4 (25.0–25.8) ^H^	266	24.6 (24.2–25.0)	**<0.001** ^e^	**<0.001** ^a^	**<0.001** ^c^
Total fat mass (kg)	315	26.6 (25.7–27.5) ^M,H^	319	24.9 (24.0–25.9) ^H^	247	23.1 (22.0–24.2)	**<0.001** ^e^	**<0.001** ^a^	**<0.001** ^c^
Fat percentage (%)	315	36.4 (35.6–37.1) ^H^	319	35.1 (34.3–35.9) ^H^	247	33.1 (32.1–34.0)	**<0.001** ^e^	**<0.001** ^a^	**<0.001** ^c^
Fat mass index (kg/m^2^)	315	9.7 (9.4–10.1) ^M,H^	319	9.1 (8.8–9.4) ^H^	247	8.4 (8.0–8.8)	**<0.001** ^e^	**<0.001** ^a^	**<0.001** ^c^
Total lean mass (kg)	315	42.3 (41.8–42.8)	319	41.8 (41.4–42.3)	247	42.3 (41.8–42.9)	0.105 ^e^	0.099 ^a^	0.195 ^c^
Lean mass index (kg/m^2^)	315	15.5 (15.4–15.6)	319	15.3 (15.2–15.5)	247	15.3 (115.2–15.5)	0.135 ^e^	**0.032** ^a^	**0.047** ^c^
ALMI (kg/m^2^)	315	6.7 (6.6–6.7)	319	6.6 (6.5–6.7)	247	6.6 (6.5–6.7)	0.237 ^e^	0.075 ^a^	0.124 ^c^
BMD									
FN BMD (g/cm^2^)	315	0.97 (0.96–0.98) ^H^	319	0.96 (0.95–0.97)	247	0.94 (0.93–0.96)	**0.034** ^e^	0.056 ^a^	0.056 ^c^
FN T score < −1	315	24.2% (76)	319	23.5% (75)	247	29.6% (73)	0.214		
FN T score ≤ −2.5	315	0.3% (1)	319	1.3% (4)	247	0.8% (2)	0.404		
*Physical performance*									
Hand grip force (N)	321	314.0 (307.2–320.8)	324	315.0 (308.6–321.5)	254	311.0 (304.0–318.1)	0.715 ^e^	0.279 ^a^	0.195 ^c^
Knee extension force (N)	282	468.8 (457.7–480.0)	271	457.8 (447.1–468.5)	230	459.7 (446.6–472.7)	0.344 ^e^	0.419 ^a^	0.442 ^c^
Jumping height (cm)	300	18.7 (18.3–19.2) ^H^	302	19.2 (18.7–19.7)	243	19.8 (19.2–20.4)	**0.013** ^e^	**0.019** ^a^	**0.013** ^c^
Walking speed (m/s)	319	2.6 (2.6–2.7)	324	2.7 (2.6–2.7)	255	2.7 (2.6–2.7)	0.443 ^e^	0.508 ^a^	0.507 ^c^
Walking distance in 6 min (m)	300	665.5 (658.5–672.5)	293	669.8 (663.2–676.3)	240	674.1 (666.0–682.1)	0.259 ^e^	0.155 ^a^	0.093 ^c^
Self-reported PA									
Leisure time PA (MET-h/d)	391	4.4 (4.0–4.8)	377	4.0 (3.6–4.3)	313	4.2 (3.8–4.5)	0.l78 ^f^	0.288 ^b^	0.285 ^d^
*Accelerometer-measured PA*									
Leisure-time MVPA (min/d)	282	42.4 (39.6–45.2)	277	41.0 (38.4–43.6)	225	46.9 (43.4–50.4)	0.054 ^f^	0.053 ^b^	**0.036** ^d^
Leisure-time step count (steps/d)	282	6812 (6495–7128) ^H^	277	6586 (6282–6892) ^H^	225	7365 (6986–7744)	**0.005** ^f^	**0.025** ^b^	**0.018** ^d^
Total MVPA (min/d)	282	48.6 (45.6–51.6)	277	48.3 (45.6–51.1)	225	53.6 (49.8–57.5)	0.117 ^f^	0.117 ^b^	0.093 ^d^
Total step count (steps/d)	282	8541 (8214–8868) ^H^	277	8492 (8179–8804) ^H^	225	9066 (8677–9455)	**0.030** ^f^	0.166 ^b^	0.169 ^d^

Values are presented as means (95% confidence intervals) or percentages (counts). M and H indicate significant difference (*p* < 0.05) from the middle and the latest menarcheal group, respectively. AAM = age at menarche; PRE = premenopausal; EPM = early perimenopausal; LPM = late perimenopausal; POST = postmenopausal; PA = physical activity; NE = no exercise; RPA = regular physical activity; CS = competitive sport; BMI = body mass index; ALMI = appendicular lean mass index; FN = femoral neck; BMD = bone mineral density; MET = metabolic equivalent; MVPA = moderate-to-vigorous physical activity. ^a^ Adjusted for PA level at age of 13–16, self-reported leisure-time PA in midlife, age, and menopausal status. ^b^ Adjusted for PA level at age 13–16, age, and menopausal status. ^c^ Adjusted for PA level at ages 13–16, self-reported leisure-time PA in midlife, age, menopausal status, number of parities, education, and hormonal contraceptive use during the 10 preceding years. ^d^ Adjusted for PA level at age 13–16, age, menopausal status, number of parities, education, and hormonal contraceptive use during the 10 preceding years. ^e^ ANOVA. ^f^ Kruskal-Wallis test. Sample sizes for both adjusted models were 299, 312, and 243 for groups reporting AAM ≤ 12 years, = 13 years, and ≥ 14 years, respectively, in height, weight, and BMI. Corresponding sample sizes for body composition measurements and BMD were 286, 295, and 228, for physical performance measurements they were 291, 299, and 234 (hand grip force), 252, 246, and 210 (knee extension force), 271, 278, and 223 (jumping height), 289, 299, and 235 (walking speed), 270, 268, and 220 (walking distance in 6 min), and for physical activity measurements they were 348, 343, and 282 (self-reported PA) and 257, 254, and 210 (accelerometer-measured PA). Statistically significant *p*-values are indicated in bold.

## Supplementary Materials

The following are available online at https://www.mdpi.com/2077-0383/9/12/3797/s1, Figure S1: Proportions of the participants in different physical activity level groups across the age categories, Table S1: Age at menarche among participants (*n* = 1081), Table S2: Proportion (*n*) of the participants reporting fractures, musculoskeletal disorders/symptoms, and anorexia nervosa diagnosis according to physical activity (PA) level at the age of 13–16, Table S3: Proportion (*n*) of the participants reporting fractures, musculoskeletal disorders/symptoms, and anorexia nervosa diagnosis according to age at menarche (AAM) groups.
