# Supplementary material for: Adolescent Sport Participation and Age at Menarche in Relation to Midlife Body Composition, Bone Mineral Density, Fitness, and Physical Activity
Source: J Clin Med. 2020 Nov 24;9(12):3797. doi: 10.3390/jcm9123797 (PMC7760316; doi:10.3390/jcm9123797)
Supplement: Supplementary file 1 [file jcm-09-03797-s001.pdf]

Journal of
Clinical Medicine

MDPI

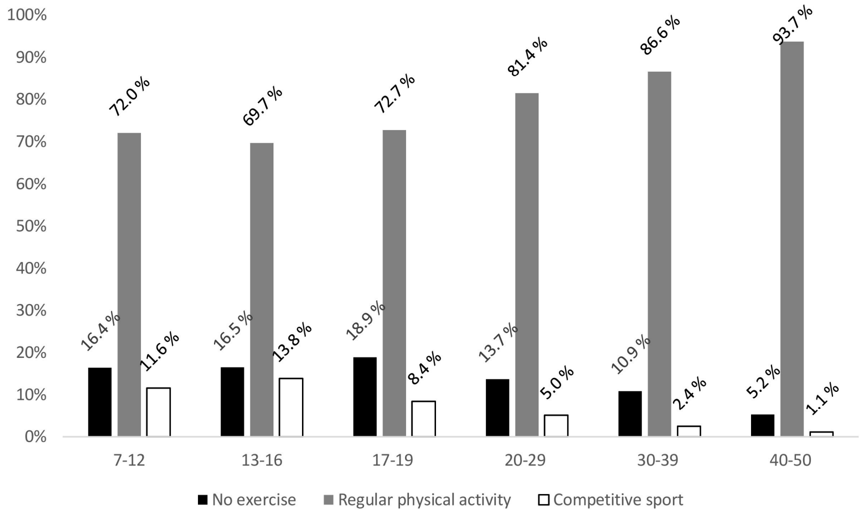

Figure S1. Proportions of the participants in different physical activity level groups across the age categories.

Table S1. Age at menarche among participants (n = 1081).

Age at menarche	Number of participants	Proportion of participants
9	2	0.2%
10	15	1.4%
11	113	10.5%
12	261	24.1%
13	377	34.9%
14	192	17.8%
15	81	7.5%
16	30	2.8%
17	8	0.7%
18	1	0.1%
19	1	0.1%

Table S2. Proportion (n) of the participants reporting fractures, musculoskeletal disorders/symptoms, and anorexia nervosa diagnosis according to physical activity (PA) level at the age of 13–16.

	Competitive sport (n = 136)	Regular PA (n = 689)	No exercise (n = 163)	p-value
Fracture	17.6% (24)	15.5% (107)	17.2% (28)	0.761
Musculoskeletal disorder/symptom	43.4% (59)	37.4% (258)	31.9% (52)	0.123
Anorexia nervosa	0.7% (1)	0.6% (4)	0.0% (0)	0.641

Data are presented as percentages (frequencies).

Table S3. Proportion (n) of the participants reporting fractures, musculoskeletal disorders/symptoms, and anorexia nervosa diagnosis according to age at menarche (AAM) groups.

	AAM ≤ 12 (n = 391)	AAM = 13 (n = 377)	AAM ≥ 14 (n = 313)	p-value
Fracture	16.6% (65)	17.2% (65)	14.4% (45)	0.571
Musculoskeletal disorder/symptom	36.6% (143)	37.9% (143)	38.0% (119)	0.901
Anorexia nervosa	0.0% (0)[a]	0.5% (2)[b]	1.3% (4)	0.069

Data are presented as percentages (frequencies). [a] n = 390; [b] n = 376.
